# Telemedicine Buprenorphine Initiation and Retention in Opioid Use Disorder Treatment for Medicaid Enrollees

**DOI:** 10.1001/jamanetworkopen.2023.36914

**Published:** 2023-10-18

**Authors:** Lindsey R. Hammerslag, Aimee Mack, Redonna K. Chandler, Laura C. Fanucchi, Daniel J. Feaster, Marc R. LaRochelle, Michelle R. Lofwall, Michael Nau, Jennifer Villani, Sharon L. Walsh, Philip M. Westgate, Svetla Slavova, Jeffery C. Talbert

**Affiliations:** 1 Institute for Biomedical Informatics, University of Kentucky College of Medicine, Lexington; 2 Division of Health Sciences, The Ohio State University Wexner Medical Center, Columbus; 3 National Institute on Drug Abuse, National Institutes of Health, Bethesda, Maryland; 4 Center on Drug and Alcohol Research, College of Medicine, University of Kentucky, Lexington; 5 Department of Public Health Sciences, University of Miami Miller School of Medicine, Miami, Florida; 6 Clinical Addiction Research & Education Unit, Boston University School of Medicine, Boston, Massachusetts; 7 Department of Biostatistics, College of Public Health, University of Kentucky, Lexington

## Abstract

**Importance:**

Early COVID-19 mitigation strategies placed an additional burden on individuals seeking care for opioid use disorder (OUD). Telemedicine provided a way to initiate and maintain transmucosal buprenorphine treatment of OUD.

**Objective:**

To examine associations between transmucosal buprenorphine OUD treatment modality (telemedicine vs traditional) during the COVID-19 public health emergency and the health outcomes of treatment retention and opioid-related nonfatal overdose.

**Design, Setting, and Participants:**

This retrospective cohort study was conducted using Medicaid claims and enrollment data from November 1, 2019, to December 31, 2020, for individuals aged 18 to 64 years from Kentucky and Ohio. Data were collected and analyzed in June 2022, with data updated during revision in August 2023.

**Exposures:**

The primary exposure of interest was the modality of the transmucosal buprenorphine OUD treatment initiation. Relevant patient demographic and comorbidity characteristics were included in regression models.

**Main Outcomes and Measures:**

There were 2 main outcomes of interest: retention in treatment after initiation and opioid-related nonfatal overdose after initiation. For outcomes measured after initiation, a 90-day follow-up period was used. The main analysis used a new-user study design; transmucosal buprenorphine OUD treatment initiation was defined as initiation after more than a 60-day gap in buprenorphine treatment. In addition, uptake of telemedicine for buprenorphine was examined, overall and within patients initiating treatment, across quarters in 2020.

**Results:**

This study included 41 266 individuals in Kentucky (21 269 women [51.5%]; mean [SD] age, 37.9 [9.0] years) and 50 648 individuals in Ohio (26 425 women [52.2%]; mean [SD] age, 37.1 [9.3] years) who received buprenorphine in 2020, with 18 250 and 24 741 people initiating buprenorphine in Kentucky and Ohio, respectively. Telemedicine buprenorphine initiations increased sharply at the beginning of 2020. Compared with nontelemedicine initiation, telemedicine initiation was associated with better odds of 90-day retention with buprenorphine in both states (Kentucky: adjusted odds ratio, 1.13 [95% CI, 1.01-1.27]; Ohio: adjusted odds ratio, 1.19 [95% CI, 1.06-1.32]) in a regression analysis adjusting for patient demographic and comorbidity characteristics. Telemedicine initiation was not associated with opioid-related nonfatal overdose (Kentucky: adjusted odds ratio, 0.89 [95% CI, 0.56-1.40]; Ohio: adjusted odds ratio, 1.08 [95% CI, 0.83-1.41]).

**Conclusions and Relevance:**

In this cohort study of Medicaid enrollees receiving buprenorphine for OUD, telemedicine buprenorphine initiation was associated with retention in treatment early during the COVID-19 pandemic. These findings add to the literature demonstrating positive outcomes associated with the use of telemedicine for treatment of OUD.

## Key Points

**Question:**

Was telemedicine initiation for buprenorphine treatment associated with changes in treatment retention or opioid-related nonfatal overdose?

**Findings:**

In this cohort study using Medicaid data on 91 914 individuals from 2020, enrollees who initiated buprenorphine treatment via telemedicine had higher odds of retention in treatment but no change in the odds of opioid-related nonfatal overdose.

**Meaning:**

This study suggests that patients may benefit from the use of telemedicine during buprenorphine initiation.

## Teaser

This cohort study of Medicaid enrollees examines associations between transmucosal buprenorphine opioid use disorder (OUD) treatment modality (telemedicine vs traditional) during the COVID-19 public health emergency and the health outcomes of treatment retention and opioid-related nonfatal overdose.

## Introduction

The COVID-19 pandemic disrupted health care delivery in the US,^1^ which may have contributed to sharp increases in drug overdose mortality.^2^ Mitigation strategies, such as stay-at-home orders and social distancing, delayed nonessential and emergency care and limited in-person medical visits. For Kentucky and Ohio, restrictions limiting access to medical care were most stringent from mid-March through the end of April 2020.^3,4,5^ These changes disproportionately affected individuals with opioid use disorder (OUD), who already faced significant barriers accessing care.^6^

Decreases in the delivery of in-person care were partially offset by an increase in telemedicine services.^7^ Telemedicine use for OUD treatment had previously been limited by regulatory and licensure restrictions, technology limitations, and reimbursement issues.^8,9^ The Coronavirus Preparedness and Response Supplemental Appropriations Act, 2020 (PL 116-123), included provisions to promote telemedicine by waiving regulatory requirements and reimbursing telemedicine visits at the same rate as in-person visits.^10^ In addition, the Substance Abuse and Mental Health Services Administration (SAMHSA) and the Drug Enforcement Administration waived licensing requirements for out-of-state clinicians as well as requirements of the 2008 Ryan Haight Act, newly permitting the use of real-time video or telephone-based communication during telemedicine initiation into buprenorphine treatment.^9,10,11,12,13,14^ As a result, increased buprenorphine telemedicine initiations were detected as early as April 2020.^15,16,17^

This study draws on data from the Healing Communities Study (HCS), funded by the National Institute on Drug Abuse and the SAMHSA. The HCS is a cluster randomized clinical trial that seeks to reduce opioid-related overdose deaths in highly affected communities across 4 states.^18^ Paramount to reducing overdose deaths is the implementation of evidence-based practices, including the provision of medications for OUD (MOUD), and improving patient retention in treatment. Telemedicine is a strategy with the potential for expanding access to MOUD, especially in rural areas lacking waivered clinicians who can prescribe buprenorphine. Preliminary evidence suggests positive outcomes for patients initiating buprenorphine treatment via telemedicine.^19,20,21^ Additional work is needed to examine outcomes for individuals who initiated telemedicine early in the COVID-19 pandemic. This work is particularly important for the HCS because the pandemic led to changes in access to care shortly before the start of the study intervention. The purpose of this study is to examine how the telemedicine regulatory changes implemented under the COVID-19 public health emergency were associated with buprenorphine treatment of OUD and whether telemedicine-associated buprenorphine initiation was associated with patient outcomes such as retention in treatment or nonfatal overdose.

This study examined the use of telemedicine for OUD care immediately prior to a buprenorphine prescription, including initial prescriptions, using 2020 data from Medicaid beneficiaries in 2 HCS states, Kentucky and Ohio. We describe general trends in the frequency of buprenorphine treatment via telemedicine, including the proportion of individuals receiving any buprenorphine via telemedicine from one quarter to the next, and analyze initiations of buprenorphine via telemedicine. We also model the association between telemedicine buprenorphine initiation and 2 outcomes: treatment retention and opioid-related nonfatal overdose. If telemedicine were associated with poorer treatment outcomes, we would expect telemedicine initiation to be associated with decreased retention and increased opioid overdose risk.

## Methods

### Data Source

Kentucky and Ohio Medicaid claims and enrollment data from November 1, 2019, through December 31, 2020, were used to construct the analytic data set. This study protocol was approved by Advarra Inc, the HCS single institutional review board. Patient consent was waived because data were deidentified. Claims data were used to identify OUD diagnoses, dispensed buprenorphine prescriptions, telemedicine services, comorbidities, and outcomes. Demographic characteristics and periods of continuous enrollment were obtained from enrollment data. This study was limited to transmucosal buprenorphine formulations approved by the US Food and Drug Administration to treat OUD (eAppendix 1 in Supplement 1). The study followed the Strengthening the Reporting of Observational Studies in Epidemiology (STROBE) reporting guideline for cohort studies.^22^

### Treatment Mode

Buprenorphine prescriptions were classified as telemedicine buprenorphine when there was a telemedicine claim with an OUD diagnosis code in the 7 days prior to the dispensing date. Telemedicine claims were identified using a combination of codes published by the Centers for Medicare & Medicaid Services,^23^ the American Academy of Physicians,^24^ a recent publication,^15^ and guidance from our state partners^25^ (eAppendix 4 in Supplement 1). If the buprenorphine prescription was an initiation prescription, meaning it was preceded by at least 60 days without buprenorphine coverage, and met the definition of telemedicine buprenorphine, then it was considered a telemedicine initiation.

### Cohort Construction

We built 2 cohorts of enrollees. The first consisted of individuals with any buprenorphine prescription dispensed in 2020. For each quarter, individuals were included if they had a buprenorphine prescription, were between 18 and 64 years of age, and had continuous Medicaid enrollment at least 60 days prior to the first buprenorphine prescription through the end of the quarter (eTable 1 in Supplement 1). Individuals observed in the first 3 quarters of 2020 (Q1-3) were also required to have continuous enrollment for the full next quarter.

The second cohort consisted of individuals with any buprenorphine initiations (prescriptions after a >60-day gap in coverage) completed in 2020. Individuals in this cohort had to meet the same enrollment criteria as those in the first cohort. Only the first buprenorphine initiation within 2020 was examined for each patient within this cohort.

### Patient Characteristics

Demographic variables (age, self-reported sex and race and ethnicity, and residential setting) were determined by the most recent Race and ethnicity were limited to the most prevalent categories labeled in the Medicaid data (Hispanic, non-Hispanic Black, and non-Hispanic White), with the remaining categories grouped into “other” (including all other races and unknown), and were analyzed because of historical disparities in access to health care and telemedicine. Medicaid enrollment records from each time period. Diagnoses for mental health conditions (eAppendix 2 in Supplement 1) and encounters indicative of opioid-related overdoses (eAppendix 3 in Supplement 1) in the 60 days prior to the first prescription were used to create flags for health history.

### Treatment Outcomes

We examined treatment outcomes among a subset of individuals with treatment initiations in Q2 or Q3 of 2020 (new-user design). This cohort was limited to patients from these 2 quarters to ensure that 90 days of follow-up were available within 2020 and because telemedicine initiation was rare in Q1 of 2020. There were 2 treatment outcomes of interest studied: (1) retention in treatment, defined as 90 days of continuous buprenorphine coverage, allowing gaps of up to 7 days, starting from the initiation date, and (2) opioid-related nonfatal overdose, defined as any medically treated encounter for opioid overdose in the first 90 days after initiation.

### Statistical Analysis

Data were collected and analyzed in June 2022, with data updated during revision in August 2023. For the cohort of all individuals with any buprenorphine prescriptions, separate χ^2^ tests were conducted each quarter to examine the association between patient demographic characteristics or comorbidities and having any telemedicine buprenorphine in a quarter. Telemedicine buprenorphine in the next quarter was visualized with a Sankey diagram depicting changes across quarters. We also identified the number of individuals who were not retained (ie, did not receive buprenorphine) in the following quarter.

For the buprenorphine initiation cohort, the percentage of patients who initiated telemedicine was first analyzed with χ^2^ tests in each quarter, stratified by patient characteristics. Analyses were conducted on a quarterly scale to simplify interpretation of trends over time, given that monthly changes in buprenorphine use during the pandemic were minimal after the initial pandemic-related disruption,^26^ and to ensure that changes over time could be tracked while complying with suppression rules for counts between 1 and 10. Weekly data were used to visualize changes in the relative frequency of telemedicine initiations throughout the course of the year. Multivariable logistic regression was used to compare the odds of having a telemedicine treatment modality initiation among patients with different demographic characteristics and baseline comorbidities. Multivariable logistic regression models were used to study the association between an outcome of interest (90-day retention in treatment or opioid-related overdose) and the modality of treatment initiation (telemedicine vs nontelemedicine), accounting for patient characteristics and the quarter of initiation. Sensitivity analyses were conducted using a shorter follow-up period, modeling retention and nonfatal overdose within the first 30 days after initiation.

Analyses for Ohio and Kentucky data were conducted separately using the same analytic approach. Results were reported as adjusted odds ratios (AORs) and 95% CIs. All analyses were conducted in SAS Enterprise Guide, version 7.1, and SAS, version 9.4 (SAS Institute Inc), and visualizations were produced in R, version 4.1.3, using RStudio, version 2022.07.1 (R Group for Statistical Computing). All *P* values were from 2-sided tests and results were deemed statistically significant at *P* < .05.

## Results

### Treatment Modality

A total of 41 266 individuals in Kentucky (19 997 men [48.5%] and 21 269 women [51.5%]; mean [SD] age, 37.9 [9.0] years) and 50 648 individuals in Ohio (24 223 men [47.8%] and 26 425 women [52.2%]; mean [SD] age, 37.1 [9.3] years) had a buprenorphine prescription in at least 1 quarter in 2020 (eTable 1 in Supplement 1). The number of individuals with buprenorphine prescriptions (eTables 2 and 3 in Supplement 1) increased from Q1 to Q4 in Kentucky (17.6% increase, from 28 111 to 33 063) and Ohio (12.4% increase, from 33 055 to 37 156). The proportion of individuals with telemedicine buprenorphine was initially low (Kentucky Q1, 14.0%; Ohio Q1, 9.8%) and increased in Q2 (Kentucky Q2, 53.9%; Ohio Q2, 38.9%) before decreasing again in both states (Kentucky Q4, 42.4%; Ohio Q4, 34.5%). Within each quarter, the proportion of individuals with telemedicine buprenorphine varied by patient demographic characteristics (eTables 2 and 3 in Supplement 1). For example, the proportion with telemedicine buprenorphine was 5% to 12% lower among non-Hispanic Black individuals compared with non-Hispanic White individuals during Q2 to Q4 in Kentucky.

**Figure 1.**
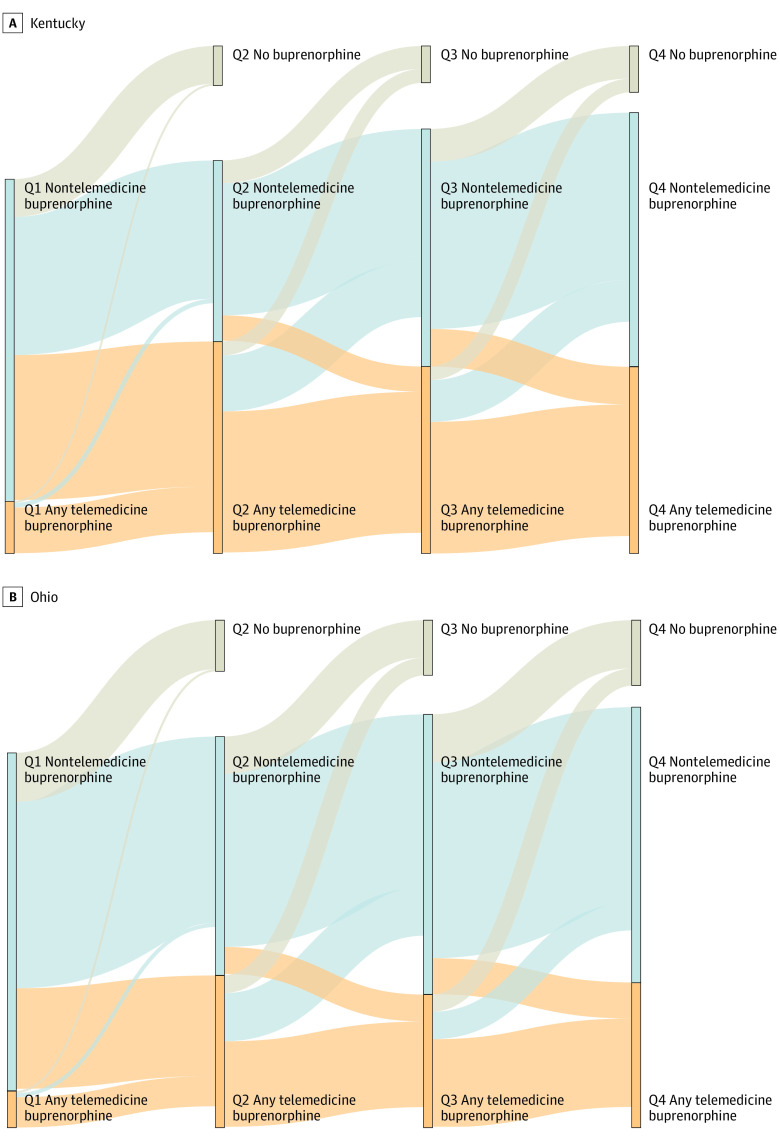
Sankey Diagram Depicting Flow of Kentucky and Ohio Patients Across Consecutive Quarters Individuals were assigned to a node (vertical bar) based on their treatment during each quarter (Q1-Q4): exclusively nontelemedicine buprenorphine (blue) or at least 1 telemedicine buprenorphine (tan). Patients who received buprenorphine in one quarter were followed up into the next quarter (connecting ribbons), with blue or tan ribbons for individuals who went on to receive buprenorphine in the next quarter and gray ribbons for individuals who did not receive buprenorphine in the next quarter. Individuals who stopped meeting inclusion criteria in the next quarter are included in the node for that quarter but not in any connecting ribbon (n = 684).

Changes in treatment modality were most common between Q1 and Q2, when 45.0% of individuals in Kentucky and 29.7% of individuals in Ohio who had nontelemedicine buprenorphine in Q1 switched to having telemedicine buprenorphine in Q2 (Figure 1; eTable 4 in Supplement 1). However, many individuals switched back to nontelemedicine buprenorphine between Q2 and Q3, with 26.4% and 31.6% of individuals in Kentucky and Ohio switching, respectively. The percentage of individuals with no buprenorphine dispensed in the next quarter was 11.7% to 17.2% for those without telemedicine and 3.8% to 12.9% for those with telemedicine.

**Figure 2.**
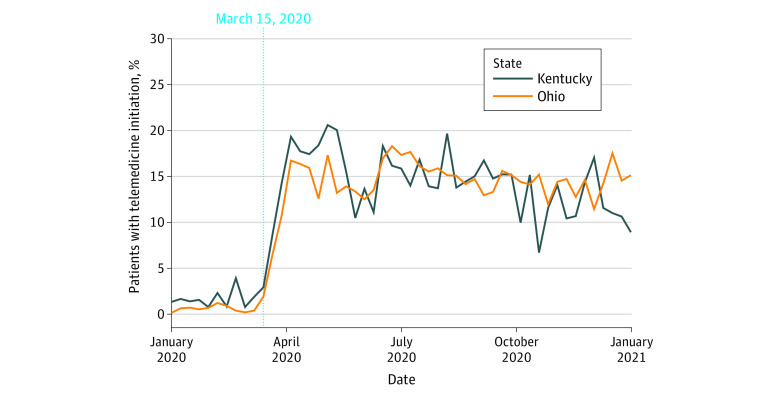
Weekly Proportion of Telemedicine Initiations, Relative to All Buprenorphine Initiations

**Figure 3.**
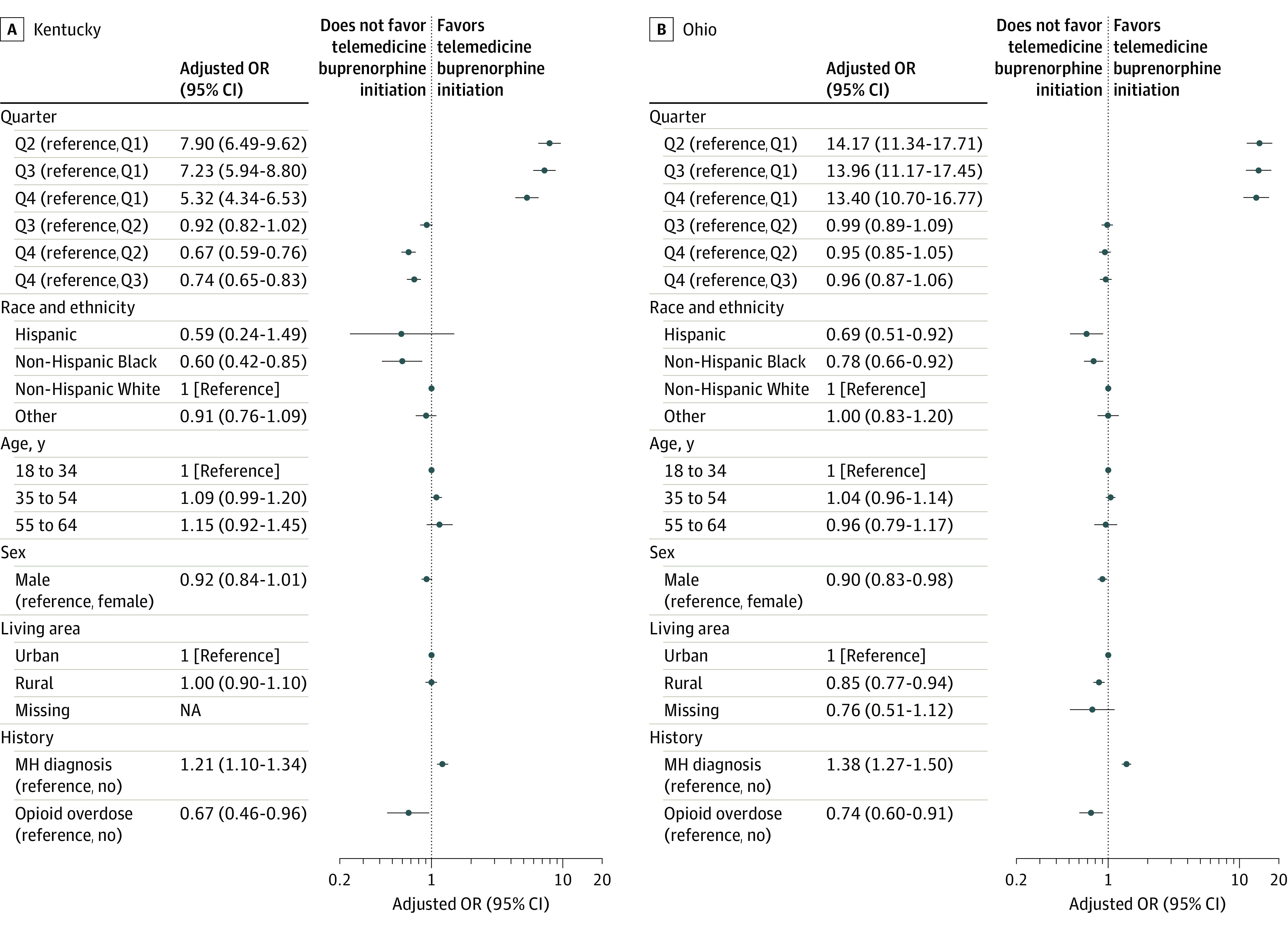
Adjusted Odds of Telemedicine Buprenorphine Initiation in Kentucky or Ohio Analysis limited to patients with buprenorphine initiations in 2020. The “other” race category includes those with a race other than Black or White as well as those with unknown race and ethnicity. MH indicates mental health; NA, not applicable; OR, odds ratio; and Q, quarter.

Within the buprenorphine initiation cohort, consisting of 18 250 individuals in Kentucky and 24 741 individuals in Ohio, the proportion with telemedicine initiation increased in both states beginning with the week of March 15, 2020 (Figure 2). On a quarterly basis, telemedicine initiations increased from Q1 (Kentucky: 2.4%, Ohio: 1.3%) to Q2 (Kentucky: 16.3%, Ohio: 15.2%) and remained elevated in subsequent quarters (eTables 5 and 6 in Supplement 1). Compared with Q1, the AORs for telemedicine initiation were at least 5.3 times higher in each of the subsequent quarters for both states (Figure 3). Telemedicine initiation was also significantly associated with race and ethnicity, sex, mental health claims, and opioid-related overdose history. For example, non-Hispanic Black individuals had lower adjusted odds of telemedicine initiation compared with non-Hispanic White individuals (Kentucky: AOR, 0.60 [95% CI, 0.42-0.85]; Ohio: AOR, 0.78 [95% CI, 0.66-0.92]). The adjusted odds of telemedicine initiation were also lower for men (Kentucky: AOR, 0.92 [95% CI, 0.84-1.01]; Ohio: AOR, 0.90 [95% CI, 0.83-0.98]), although the difference in Kentucky was nonsignficant, and for individuals with a prior opioid-related overdose (Kentucky: AOR, 0.67 [95% CI, 0.46-0.96]; Ohio: AOR, 0.74 [95% CI, 0.60-0.91]).

### Retention

**Figure 4.**
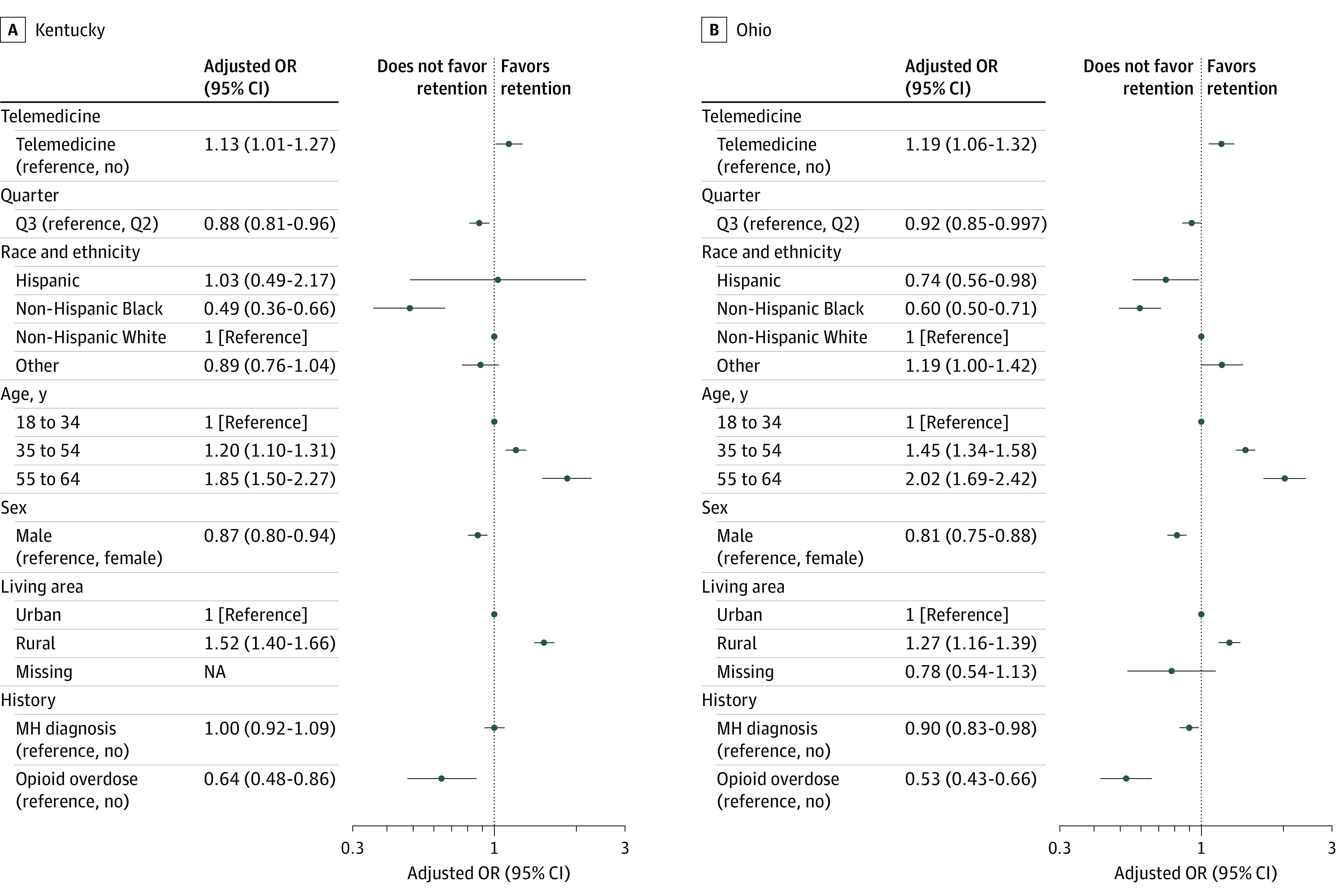
Telemedicine Initiation and the Adjusted Odds of Retention in Kentucky or Ohio Analysis limited to patients with buprenorphine initiations in quarter 2 (Q2) or Q3 of 2020. The “other” race category includes those with a race other than Black or White as well as those with unknown race and ethnicity. MH indicates mental health; NA, not applicable; OR, odds ratio; and Telemedicine, telemedicine initiation.

Among the subset of individuals with buprenorphine initiation in Q2 or Q3, the proportion with continuous retention for at least 90 days after initiation was 45.0% in Kentucky and 28.5% in Ohio (eTable 7 in Supplement 1). Telemedicine initiation was associated with higher odds of retention in the unadjusted analysis (Kentucky: OR, 1.16 [95% CI, 1.03-1.29]; Ohio: OR, 1.19 [95% CI, 1.07-1.32]). Telemedicine was also associated with retention in the adjusted analysis, with AORs of 1.13 (95% CI, 1.01-1.27) in Kentucky and 1.19 (95% CI, 1.06-1.32) in Ohio (Figure 4). The odds of retention in both states were also significantly associated with initiation quarter, race and ethnicity, age, sex, rural status, and opioid-related overdose history. For example, the adjusted odds of retention were lower for non-Hispanic Black individuals compared with non-Hispanic White individuals (Kentucky: AOR, 0.49 [95% CI, 0.36-0.66]; Ohio: AOR, 0.60 [95% CI, 0.50-0.71]), men (Kentucky: AOR, 0.87 [95% CI, 0.80-0.94]; Ohio: AOR, 0.81 [95% CI, 0.75-0.88]) and individuals with a prior opioid-related overdose (Kentucky: AOR, 0.64 [95% CI, 0.48-0.86]; Ohio: AOR, 0.53 [95% CI, 0.43-0.66]). In the sensitivity analysis, 69.6% of individuals in Kentucky and 48.1% of individuals in Ohio were retained for 30 days after initiation (eTable 8 in Supplement 1). The association between telemedicine and 30-day retention was similar in the unadjusted (Kentucky: OR, 1.34 [95% CI, 1.18-1.52]; Ohio: OR, 1.28 [95% CI, 1.16-1.42]) and adjusted (Kentucky: AOR, 1.33 [95% CI, 1.17-1.51]; Ohio: AOR, 1.28 [95% CI, 1.16-1.41]) sensitivity analyses (eFigure 1 in Supplement 1).

### Nonfatal Opioid Overdose

**Figure 5.**
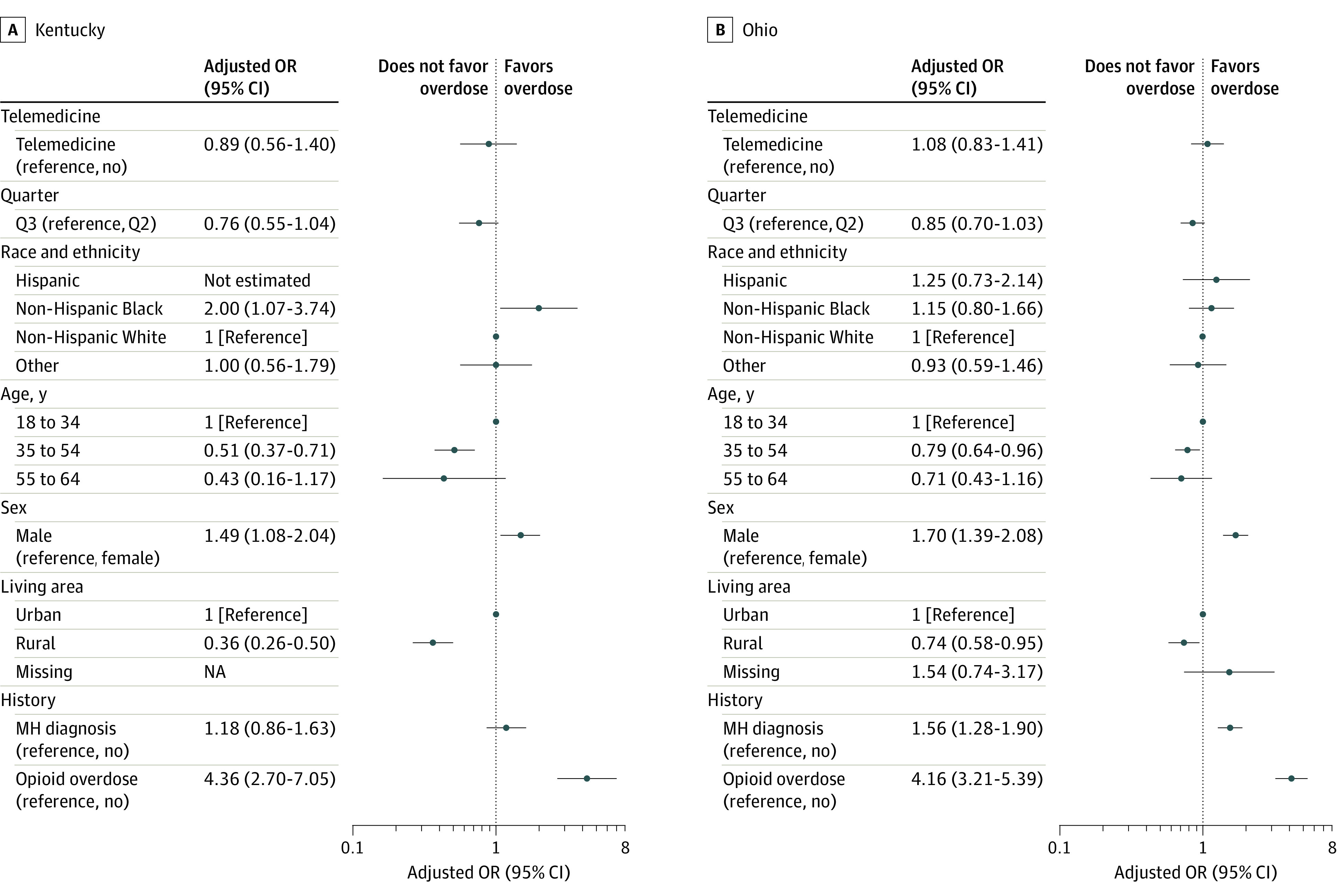
Telemedicine Initiation and the Adjusted Odds of Opioid-Related Nonfatal Overdose in Kentucky or Ohio Analysis limited to patients with buprenorphine initiations in quarter 2 (Q2) or Q3 of 2020. The “other” race category includes those with a race other than Black or White as well as those with unknown race and ethnicity. MH indicates mental health; NA, not applicable; OR, odds ratio; and Telemedicine, telemedicine initiation.

Among individuals with buprenorphine initiation in Q2 or Q3, the prevalence of opioid-related nonfatal overdose in the 90 days after initiation was 1.8% in Kentucky and 3.6% in Ohio (eTable 9 in Supplement 1). Telemedicine initiation was not associated with opioid-related overdose in either the unadjusted (Kentucky: OR, 0.83 [95% CI, 0.53-1.30]; Ohio: OR, 1.07 [95% CI, 0.83-1.39]) or adjusted analyses, with AORs of 0.89 (95% CI, 0.56-1.40) in Kentucky and 1.08 (95% CI, 0.83-1.41) in Ohio (Figure 5). The only factors that were significantly associated with opioid-related overdose in both states were age, sex, rural status, and opioid-related overdose history. For example, the adjusted odds of opioid-related overdose were higher for men (Kentucky: AOR, 1.49 [95% CI, 1.08-2.04]; Ohio: AOR, 1.70 [95% CI, 1.39-2.08]) and individuals with a prior opioid-related overdose (Kentucky: AOR, 4.36 [95% CI, 2.70-7.05]; Ohio: AOR, 4.16 [95% CI, 3.21-5.39]). In the sensitivity analysis, 0.7% of individuals in Kentucky and 1.4% of individuals in Ohio experienced a nonfatal overdose within 30 days of initiation (eTable 10 in Supplement 1). As in the main analysis, there was no association of telemedicine induction with the unadjusted (Kentucky: OR, 0.82 [95% CI, 0.40-1.65]; Ohio: OR, 0.85 [95% CI, 0.55-1.32]) or adjusted (Kentucky: AOR, 0.84 [95% CI, 0.41-1.71]; Ohio: AOR, 0.87 [95% CI, 0.56-1.36]) odds of overdose (eFigure 2 in Supplement 1).

## Discussion

We identified marked increases in telemedicine delivery of buprenorphine for Ohio and Kentucky Medicaid patients, corresponding to more permissive telehealth regulations associated with the COVID-19 pandemic. The proportion of telemedicine buprenorphine initiations increased similarly in Kentucky and Ohio between the first 2 quarters of 2020, and telemedicine initiation was associated with better retention in buprenorphine treatment in both states. Telemedicine initiation was not associated with opioid-related nonfatal overdose, although overall rates of opioid-related overdose were low and the power to detect a difference was limited.

We identified disparities by race and ethnicity in both receipt of telemedicine initiation and retention, with non-Hispanic Black individuals having lower odds of telemedicine initiation and approximately half the odds of being retained in buprenorphine treatment at 90 days, relative to non-Hispanic White individuals. These data are consistent with other recent studies^27^: one found that disparities in substance use disorder care widened during the early pandemic,^28^ and there were also disparities in the type of telemedicine (video or telephone only) care that patients received.^29^

Our research suggests that individuals who received telemedicine visits for OUD care in the week prior to buprenorphine initiation were not more likely to experience opioid overdose. In fact, patients who initiated buprenorphine treatment via telemedicine were more likely to be retained in treatment than those without telemedicine initiation. These results are consistent with pre–COVID-19 studies reporting better retention when telemedicine visits were included in care.^19,20,21^ They are also consistent with recent studies examining Veterans Health Administration^29^ and Medicare^16^ populations. In the present study, telemedicine initiation was more common among individuals with behavioral health claims in the months prior to initiation. This finding may be associated with clinicians reporting hesitancy around initiating unfamiliar patients using telemedicine,^30^ or it could reflect some unmeasured aspect of clinician decision-making. For example, some clinicians may be uncomfortable with using telemedicine specifically for buprenorphine treatment of OUD due to regulatory barriers and complexity,^31^ and pandemic-related efforts to improve telemedicine access may have been challenging to interpret, particularly as telemedicine policies evolved later in the pandemic. In 2021, for example, Kentucky’s Board of Medical Licensure issued an opinion that acknowledged the utility of telemedicine for buprenorphine treatment while also expressing that this modality “is not appropriate for satisfying the standards of all components of a treatment program, particularly in regard to monitoring components.”^32^ State legislation enacted at the end of 2021 in Ohio defined clinicians who may provide telemedicine services and removed the requirement of an in-person visit for patients receiving MOUD. Laws such as this may bolster clinicians’ confidence in the effectiveness of telemedicine for substance use treatment.^33^

These results offer important insights for states with a high burden of OUD looking to policies and methods to reduce barriers to treatment. The HCS selected states with high rates of opioid-related overdose (Kentucky, Ohio, New York, and Massachusetts). As telemedicine increased with the start of the pandemic, the HCS team began reviewing the use of telemedicine MOUD initiation to understand the association of the policy changes with the study outcomes. Our results show that telemedicine was associated with increased access to MOUD and improved retention. This finding may be especially valuable for improving MOUD access in states such as Kentucky, which has historically had restrictive buprenorphine access policies^34^ and restrictive methadone regulations, as well as a large rural population.^35^ Telemedicine may also increase access in underserved areas, adding to the workforce for behavioral health services in rural areas. In the present study, however, rural Ohio residents were less likely to initiate buprenorphine with telemedicine, and rural residents in both states had better patient outcomes. The association between urbanization and MOUD outcomes varies across states,^36^ and there may be heterogeneity in the association of telemedicine with rural buprenorphine access.

Our results also provide insights about sustaining telemedicine care for MOUD after the pandemic emergency regulations end. The declaration of the national public health emergency allowed federal and state regulation changes to support increased access to MOUD and telemedicine buprenorphine initiation. Beyond regulation changes, reimbursement issues are key factors limiting telemedicine expansion. The Centers for Medicare & Medicaid Services issued guidance allowing states to provide reimbursement parity for telemedicine services compared with in-person services. Most states (n = 42) approved Medicaid parity requirements for general telemedicine visits, but only 17 approved these requirements for mental health visits, and fewer states approved commercial insurance parity requirements.^37^ Although telemedicine visits may be more convenient, bypassing the need to take time off or find transportation to receive treatment, payers are reluctant to provide parity if telemedicine is viewed as inferior to in-person care or is associated with potential harms. Our findings provide some support for parity requirements by demonstrating that telemedicine was associated with improved access and improved treatment retention.

### Limitations

There are several limitations to our analysis. For example, there may have been unmeasured confounders, such as perceived patient stability, that influenced our findings. Follow-up studies using causal inference or examining patient and clinician experiences and preferences would be valuable for understanding who is offered telemedicine during buprenorphine initiation. Given the changes in telemedicine policies and infrastructure in the years after 2020,^32,33^ it will be important for future studies to examine whether telemedicine initiation was associated with better retention beyond 2020. Similarly, the results from the present study may not be generalizable beyond the Medicaid populations in Kentucky and Ohio. We required continuous enrollment in Medicaid, which may have biased the sample away from negative outcomes by excluding incarcerated individuals and those who died. In addition, the continuous enrollment requirement would have affected individuals differently starting in March 2020 because Medicaid agencies paused unenrollment based on eligibility during the COVID-19 public health emergency. Because Medicaid data were not linked to emergency services or death records, this study considered only medically treated overdose. Overdose was a rare outcome, and so the power to detect an association between telemedicine and overdose may have been limited. Estimated associations with telemedicine may also have been biased toward the null if changing billing guidelines led to the misclassification of telemedicine services. Finally, we did not distinguish between different models of telemedicine (eg, telephone and video), and some groups may have had less access to technology used in real-time video telemedicine. Telephone-only telemedicine may be associated with lower odds of retention compared with video telemedicine, and non-Hispanic Black individuals may have lower use of video telemedicine.^29,38^ Future work examining audio-only vs audiovisual telemedicine will be valuable because audio-only telemedicine may be more accessible, but it is unclear whether evolving regulations around reimbursement parity will also lead to parity for audio-only care.^39^

## Conclusions

Mitigation strategies enacted during the COVID-19 pandemic placed an additional burden on individuals seeking care for OUD. Telemedicine provided a way to initiate and/or continue OUD treatment with buprenorphine. In this cohort study, we examined how telemedicine regulatory changes implemented under the COVID-19 public health emergency were associated with buprenorphine initiation, buprenorphine retention, and management of OUD. The number of individuals receiving buprenorphine for treatment of OUD increased throughout 2020. The proportion of buprenorphine prescriptions supported by telemedicine increased and then decreased, stabilizing above prepandemic levels. Individuals using telemedicine initiation were more likely to achieve 90-day retention with buprenorphine. Opioid-related nonfatal overdose was not associated with telemedicine initiation. Our results suggest that telemedicine is a strategy associated with increased access to MOUD and improved retention. We did not find any association between telemedicine and nonfatal overdose, although additional work is needed to examine whether this finding would apply to individuals other than continuously enrolled Medicaid beneficiaries in Kentucky and Ohio. This work addresses important questions around the growing interest in continuing the use of telemedicine to support treatment and management of OUD after the COVID-19 public health emergency ends^8,40,41,42,43^ and provides insights into the utility of telemedicine to improve patient outcomes.

## Notes

Supplement 1. **eTable 1.** Exclusion Steps Followed for the Construction of the Two Cohorts Used in the Current Study **eTable 2.** Demographic Information for Patients in the Kentucky Cohort of All Individuals Receiving Buprenorphine, With the Proportion That Received Any Telemedicine Buprenorphine Within Each Quarter **eTable 3.** Demographic Information for Patients in the Ohio Cohort of All Individuals Receiving Buprenorphine, With the Proportion That Received Any Telemedicine Buprenorphine Within Each Quarter **eTable 4.** Sankey Diagram Data From Figure 1, Depicting the Change in Treatment Modalities From One Quarter to the Next **eTable 5.** Frequency of Telemedicine Initiations, Relative to All Buprenorphine Initiations, in Kentucky **eTable 6.** Frequency of Telemedicine Initiations, Relative to All Buprenorphine Initiations, in Ohio **eTable 7.** Percentage of Individuals Achieving 90-Day Retention in Kentucky and Ohio **eTable 8.** Sensitivity Analysis Examining Percentage of Individuals Achieving 30-Day Retention in Kentucky and Ohio **eTable 9.** Percentage of Individuals With an Opioid-Related Overdose During the 90-Day Follow-up Period in Kentucky and Ohio **eTable 10.** Sensitivity Analysis Examining Percentage of Individuals With an Opioid-Related Overdose During the 30-Day Follow-up Period in Kentucky and Ohio **eFigure 1.** Adjusted Odds of Retention Over the First 30 Days After Initiation in Kentucky (A) or Ohio (B) **eFigure 2.** Adjusted Odds of Opioid-Related Nonfatal Overdose in the First 30 Days After Initiation in Kentucky (A) or Ohio (B) **eAppendix 1.** National Drug Codes (NDC) Used to Identify Transmucosal Buprenorphine FDA-Approved for the Treatment of OUD **eAppendix 2.** Diagnosis Codes for Mental Health Conditions (The Medicaid Outcomes Distributed Research Network (MODRN) et al, 2021) **eAppendix 3.** Diagnosis Codes for Opioid-Related Overdoses (Poisonings) **eAppendix 4.** Protocol for Identifying Telemedicine Visits

Supplement 2.
Data Sharing Statement
