# Supplementary material for: Telemedicine Buprenorphine Initiation and Retention in Opioid Use Disorder Treatment for Medicaid Enrollees
Source: JAMA Netw Open. 2023 Oct 18;6(10):e2336914. doi: 10.1001/jamanetworkopen.2023.36914 (PMC10585416; doi:10.1001/jamanetworkopen.2023.36914)
Supplement: Supplement 1. — eTable 1. Exclusion Steps Followed for the Construction of the Two Cohorts Used in the Current Study eTable 2. Demographic Information for Patients in the Kentucky Cohort of All Individuals Receiving Buprenorphine, With the Proportion That Received Any Telemedicine Buprenorphine Within Each Quarter eTable 3. Demographic Information for Patients in the Ohio Cohort of All Individuals Receiving Buprenorphine, With the Proportion That Received Any Telemedicine Buprenorphine Within Each Quarter eTable 4. Sankey Diagram Data From Figure 1, Depicting the Change in Treatment Modalities From One Quarter to the Next eTable 5. Frequency of Telemedicine Initiations, Relative to All Buprenorphine Initiations, in Kentucky eTable 6. Frequency of Telemedicine Initiations, Relative to All Buprenorphine Initiations, in Ohio eTable 7. Percentage of Individuals Achieving 90-Day Retention in Kentucky and Ohio eTable 8. Sensitivity Analysis Examining Percentage of Individuals Achieving 30-Day Retention in Kentucky and Ohio eTable 9. Percentage of Individuals With an Opioid-Related Overdose During the 90-Day Follow-up Period in Kentucky and Ohio eTable 10. Sensitivity Analysis Examining Percentage of Individuals With an Opioid-Related Overdose During the 30-Day Follow-up Period in Kentucky and Ohio eFigure 1. Adjusted Odds of Retention Over the First 30 Days After Initiation in Kentucky (A) or Ohio (B) eFigure 2. Adjusted Odds of Opioid-Related Nonfatal Overdose in the First 30 Days After Initiation in Kentucky (A) or Ohio (B) eAppendix 1. National Drug Codes (NDC) Used to Identify Transmucosal Buprenorphine FDA-Approved for the Treatment of OUD eAppendix 2. Diagnosis Codes for Mental Health Conditions (The Medicaid Outcomes Distributed Research Network (MODRN) et al, 2021) eAppendix 3. Diagnosis Codes for Opioid-Related Overdoses (Poisonings) eAppendix 4. Protocol for Identifying Telemedicine Visits [file jamanetwopen-e2336914-s001.pdf]

Supplementary Online Content

Hammerslag LR, Mack A, Chandler RK, et al. Telemedicine buprenorphine initiation and retention in opioid use disorder treatment for Medicaid enrollees. *JAMA Netw Open.* 2023;6(10):e2336914. doi:10.1001/jamanetworkopen.2023.36914

eTable 1. Exclusion Steps Followed for the Construction of the Two Cohorts Used in the Current Study
eTable 2. Demographic Information for Patients in the Kentucky Cohort of All Individuals Receiving Buprenorphine, With the Proportion That Received Any Telemedicine Buprenorphine Within Each Quarter
eTable 3. Demographic Information for Patients in the Ohio Cohort of All Individuals Receiving Buprenorphine, With the Proportion That Received Any Telemedicine Buprenorphine Within Each Quarter
eTable 4. Sankey Diagram Data From Figure 1, Depicting the Change in Treatment Modalities From One Quarter to the Next
eTable 5. Frequency of Telemedicine Initiations, Relative to All Buprenorphine Initiations, in Kentucky
eTable 6. Frequency of Telemedicine Initiations, Relative to All Buprenorphine Initiations, in Ohio
eTable 7. Percentage of Individuals Achieving 90-Day Retention in Kentucky and Ohio
eTable 8. Sensitivity Analysis Examining Percentage of Individuals Achieving 30-Day Retention in Kentucky and Ohio
eTable 9. Percentage of Individuals With an Opioid-Related Overdose During the 90-Day Follow-up Period in Kentucky and Ohio
eTable 10. Sensitivity Analysis Examining Percentage of Individuals With an Opioid-Related Overdose During the 30-Day Follow-up Period in Kentucky and Ohio
eFigure 1. Adjusted Odds of Retention Over the First 30 Days After Initiation in Kentucky (A) or Ohio (B)
eFigure 2. Adjusted Odds of Opioid-Related Nonfatal Overdose in the First 30 Days After Initiation in Kentucky (A) or Ohio (B)
eAppendix 1. National Drug Codes (NDC) Used to Identify Transmucosal Buprenorphine FDA-Approved for the Treatment of OUD
eAppendix 2. Diagnosis Codes for Mental Health Conditions (the Medicaid Outcomes Distributed Research Network (MODRN) et al, 2021)
eAppendix 3. Diagnosis Codes for Opioid-Related Overdoses (Poisonings)
eAppendix 4. Protocol for Identifying Telemedicine Visits

This supplementary material has been provided by the authors to give readers additional information about their work.

© 2023 Hammerslag LR et al. *JAMA Network Open.*

eTable 1. Exclusion Steps Followed for the Construction of the Two Cohorts Used in the Current Study

State, Exclusion Criteria Step	Q1 2020 Individuals	Q2 2020 Individuals	Q3 2020 Individuals	Q4 2020 Individuals	Total Individuals[1]
**Kentucky**					
1. All enrollees in quarter.	1,428,993	1,525,069	1,633,315	1,682,537	1,766,014
2. Include only individuals receiving buprenorphine for opioid use disorder in quarter.	29,663	30,989	33,504	34,210	43,427
3. Include only individuals aged 18-64 that have continuous Medicaid 60 days prior to index Rx and through end of quarter.	28,277	29,676	32,310	33,063	41,754
4. For quarter 1 through quarter 3, include only enrollees with continuous Medicaid for the entire next quarter. This is the final dataset for Cohort 1.	28,111	29,498	31,857	33,063	41,266
5. Include only buprenorphine initiations, with at least a 60-day gap in coverage before the index buprenorphine prescription in the quarter.	5,042	4,584	5,428	4,917	18,250
6. Include only individuals that did not have an initiation in a prior quarter within 2020. This is the final dataset for Cohort 2.	5,042	4,341	4,831	4,036	18,250
**Ohio**					
1. All enrollees in quarter.	2,840,931	2,901,550	2,993,285	3,069,739	3,203,626
2. Include only individuals receiving buprenorphine for opioid use disorder in quarter.	35,478	36,603	38,109	38,250	53,463
3. Include only individuals aged 18-64 that have continuous Medicaid 60 days prior to index Rx and through end of quarter.[2]	33,285	34,763	36,693	37,156	50,965
4. For quarter 1 through quarter 3, include only enrollees with continuous Medicaid for the entire next quarter. This is the final dataset for Cohort 1.	33,055	34,517	36,470	37,156	50,648
5. Include only buprenorphine initiations, with at least a 60-day gap in coverage before the index buprenorphine prescription in the quarter.	6,935	6,589	7,300	7,177	24,741
6. Include only individuals that did not have an initiation in a prior quarter within 2020. This is the final dataset for Cohort 2.	6,935	6,131	6,197	5,478	24,741

[1]The count of total individuals represents the deduplicated total number of individuals that were included in at least one quarter for each step.

[2]In Ohio, age information is only available for those with enrollment in a given month and so enrollees were required to have a full 3 months of enrollment rather than just the 60 days post-index.

© 2023 Hammerslag LR et al. *JAMA Network Open.*

eTable 2. Demographic Information for Patients in the Kentucky Cohort of All Individuals Receiving Buprenorphine, With the Proportion That Received Any Telemedicine Buprenorphine Within Each Quarter

**Kentucky**	**Q1 2020**		**Q2 2020**		**Q3 2020**		**Q4 2020**	
**Total N (% with any telemedicine buprenorphine dispensation)**	**N (%)**	***p* value**	**N (%)**	***p* value**	**N (%)**	***p* value**	**N (%)**	***p* value**
**Patient was inducted (>60 days without Buprenorphine coverage before first prescription)**		< 0.001		< 0.001		< 0.001		< 0.001
Yes	5,042 (11.7%)		4,584 (32.4%)		5,428 (28.1%)		4,917 (26.7%)	
No	23,069 (14.5%)		24,914 (57.9%)		26,429 (47.3%)		28,146 (45.1%)	
**Race/Ethnicity**		0.34		0.05		< 0.001		< 0.001
Hispanic	64 (20.3%)		67 (58.2%)		81 (25.9%)		101 (34.7%)	
Non-Hispanic Black	400 (13.8%)		429 (48.7%)		484 (32.2%)		542 (30.1%)	
Non-Hispanic White	25,641 (14.0%)		26,902 (54.1%)		28,991 (44.5%)		29,970 (42.8%)	
Others[1]	2,006 (13.2%)		2,100 (52.3%)		2,301 (41.4%)		2,450 (39.9%)	
**Age group**		0.16		0.37		0.21		0.07
18-34	10,350 (13.6%)		10,727 (53.4%)		11,352 (43.4%)		11,492 (42.2%)	
35-54	16,020 (14.3%)		16,928 (54.3%)		18,480 (44.5%)		19,456 (42.7%)	
55-64	1,741 (13.1%)		1,843 (53.9%)		2,025 (43.7%)		2,115 (40.1%)	
**Sex**		0.01		< 0.001		< 0.001		< 0.001
Male	12,841 (13.4%)		13,617 (52.3%)		14,837 (42.3%)		15,601 (40.4%)	
Female	15,270 (14.5%)		15,881 (55.3%)		17,020 (45.6%)		17,462 (44.1%)	
**Living Area**		< 0.001		< 0.001		< 0.001		< 0.001
Urban	8,463 (16.0%)		9,069 (51.5%)		9,995 (37.5%)		10,414 (36.0%)	
Rural	19,648 (13.1%)		20,429 (55.0%)		21,862 (47.0%)		22,649 (45.3%)	
Missing Urban/Rural Category	0 (0.0%)		0 (0.0%)		0 (0.0%)		0 (0.0%)	
**Comorbidities during 60-day lookback**		< 0.001		0.21		0.001		< 0.001
Any Mental Health Codes	9,536 (16.6%)		10,447 (54.4%)		11,329 (45.2%)		12,049 (44.7%)	
No Mental Health Codes	18,575 (12.6%)		19,051 (53.6%)		20,528 (43.4%)		21,014 (41.0%)	
**Opioid-related overdose during the 60-day lookback**		0.95		< 0.001		< 0.001		< 0.001
Yes	134 (14.2%)		182 (29.7%)		241 (26.1%)		181 (28.7%)	
No	27,977 (14.0%)		29,316 (54.1%)		31,616 (44.2%)		32,882 (42.4%)	
**Total, N**	28,111 (14.0%)		29,498 (53.9%)		31,857 (44.0%)		33,063 (42.4%)	

[1]The “Others” race category includes those with a race other than White or Black as well as those with unknown race and ethnicity.

© 2023 Hammerslag LR et al. *JAMA Network Open.*

eTable 3. Demographic Information for Patients in the Ohio Cohort of All Individuals Receiving Buprenorphine, With the Proportion That Received Any Telemedicine Buprenorphine Within Each Quarter

Ohio	Q1 2020		Q2 2020		Q3 2020		Q4 2020	
**Total N (% with any telemedicine buprenorphine dispensation)**	**N (%)**	***p* value**	**N (%)**	***p* value**	**N (%)**	***p* value**	**N (%)**	***p* value**
**Patient was inducted (>60 days without Buprenorphine coverage before first prescription)**		0.12		< 0.001		< 0.001		< 0.001
Yes	6,935 (9.3%)		6,589 (28.8%)		7,300 (25.3%)		7,177 (27.5%)	
No	26,120 (9.9%)		27,928 (41.3%)		29,170 (34.0%)		29,979 (36.2%)	
**Race/Ethnicity**		0.03		0.52		0.21		0.83
Hispanic	661 (12.1%)		694 (40.8%)		755 (30.9%)		797 (33.9%)	
Non-Hispanic Black	1,762 (8.2%)		1,749 (39.3%)		1,835 (34.3%)		1,946 (34.2%)	
Non-Hispanic White	29,045 (9.8%)		30,417 (38.9%)		32,101 (32.2%)		32,571 (34.5%)	
Others[1]	1,587 (9.6%)		1,657 (37.7%)		1,779 (31.6%)		1,842 (35.4%)	
**Age group**		0.92		0.21		0.002		0.13
18-34	13,938 (9.7%)		14,424 (39.4%)		15,041 (32.8%)		14,903 (35.1%)	
35-54	16,757 (9.8%)		17,664 (38.7%)		18,825 (32.2%)		19,603 (34.2%)	
55-64	2,360 (9.9%)		2,429 (37.8%)		2,604 (29.3%)		2,650 (33.7%)	
**Sex**		0.004		< 0.001		< 0.001		< 0.001
Male	14,711 (9.2%)		15,605 (37.3%)		16,597 (31.3%)		17,088 (32.8%)	
Female	18,344 (10.2%)		18,912 (40.3%)		19,873 (33.0%)		20,068 (36.0%)	
**Living Area**		0.21		0.20		< 0.001		< 0.001
Urban	23,846 (9.9%)		24,781 (39.0%)		26,147 (33.2%)		26,709 (36.1%)	
Rural	8,813 (9.3%)		9,373 (39.0%)		9,954 (29.8%)		10,095 (30.6%)	
Missing Urban/Rural Category	396 (9.3%)		363 (34.4%)		369 (27.9%)		352 (28.4%)	
**Comorbidities during 60-day lookback**		< 0.001		< 0.001		< 0.001		< 0.001
Any Mental Health Codes	13,722 (12.1%)		14,630 (45.7%)		15,462 (37.9%)		16,199 (40.4%)	
No Mental Health Codes	19,333 (8.1%)		19,887 (33.9%)		21,008 (28.0%)		20,957 (29.9%)	
**Opioid-related overdose during the 60-day lookback**		0.12		0.13		0.10		0.03
Yes	536 (11.8%)		545 (35.8%)		711 (29.4%)		583 (30.2%)	
No	32,519 (9.7%)		33,972 (39.0%)		35,759 (32.3%)		36,573 (34.6%)	
**Total, N**	33,055 (9.8%)		34,517 (38.9%)		36,470 (32.2%)		37,156 (34.5%)	

[1]The “Others” race category includes those with a race other than White or Black as well as those with unknown race and ethnicity.

© 2023 Hammerslag LR et al. *JAMA Network Open.*

eTable 4. Sankey Diagram Data From Figure 1, Depicting the Change in Treatment Modalities From One Quarter to the Next

		Treatment in a Given Quarter (Vertical Bars)				Treatment in the Next Quarter (Connecting Ribbons)			
**State**	**Quarter**	**Treatment During Quarter**	**Bar Color**	**N Patients**	**% of Treated Individuals**	**Non-Telemedicine Buprenorphine (Blue)**	**Telemedicine Buprenorphine (Tan)**	**No Buprenorphine (Gray)**	**Ineligible (No Line)**
KY	1	Non-Tele Bup	Blue	24,179	86.0%	10,371 (42.9%)	10,877 (45.0%)	2,822 (11.7%)	109 (0.5%)
KY	1	Any Tele Bup	Tan	3,932	14.0%	334 (8.5%)	3,427 (87.2%)	148 (3.8%)	23 (0.6%)
KY	2	Non-Tele Bup	Blue	13,594	46.1%	9,914 (72.9%)	1,896 (13.9%)	1,710 (12.6%)	74 (0.5%)
KY	2	Any Tele Bup	Tan	15,904	53.9%	4,195 (26.4%)	10,580 (66.5%)	1,051 (6.6%)	78 (0.5%)
KY	2	No Bup	Gray	2,970	N/A[1]	N/A[2]	N/A[2]	N/A[2]	N/A[2]
KY	3	Non-Tele Bup	Blue	17,828	56.0%	12,559 (70.4%)	2,822 (15.8%)	2,447 (13.7%)	0 (0.0%)
KY	3	Any Tele Bup	Tan	14,029	44.0%	3,125 (22.3%)	9,875 (70.4%)	1,029 (7.3%)	0 (0.0%)
KY	3	No Bup	Gray	2,761	N/A[1]	N/A[2]	N/A[2]	N/A[2]	N/A[2]
KY	4	Non-Tele Bup	Blue	19,055	57.6%	N/A[3]	N/A[3]	N/A[3]	N/A[3]
KY	4	Any Tele Bup	Tan	14,008	42.4%	N/A[3]	N/A[3]	N/A[3]	N/A[3]
KY	4	No Bup	Blue	3,476	N/A[1]	N/A[3]	N/A[3]	N/A[3]	N/A[3]
OH	1	Non-Tele Bup	Tan	29,829	90.2%	16,463 (55.2%)	8,868 (29.7%)	4,321 (14.5%)	177 (0.6%)
OH	1	Any Tele Bup	Blue	3,226	9.8%	343 (10.6%)	2,674 (82.9%)	190 (5.9%)	19 (0.6%)
OH	2	Non-Tele Bup	Tan	21,077	61.1%	15,285 (72.5%)	2,382 (11.3%)	3,291 (15.6%)	119 (0.6%)
OH	2	Any Tele Bup	Gray	13,440	38.9%	4,246 (31.6%)	7,550 (56.2%)	1,572 (11.7%)	72 (0.5%)
OH	2	No Bup	Blue	4,511	N/A[1]	N/A[2]	N/A[2]	N/A[2]	N/A[2]
OH	3	Non-Tele Bup	Tan	24,721	67.8%	17,283 (69.9%)	3,182 (12.9%)	4,245 (17.2%)	11 (0.0%)
OH	3	Any Tele Bup	Gray	11,749	32.2%	2,421 (20.6%)	7,804 (66.4%)	1,520 (12.9%)	*
OH	3	No Bup	Blue	4,863	N/A[1]	N/A[2]	N/A[2]	N/A[2]	N/A[2]
OH	4	Non-Tele Bup	Tan	24,335	65.5%	N/A[3]	N/A[3]	N/A[3]	N/A[3]
OH	4	Any Tele Bup	Blue	12,821	34.5%	N/A[3]	N/A[3]	N/A[3]	N/A[3]
OH	4	No Bup	Tan	5,765	N/A[1]	N/A[3]	N/A[3]	N/A[3]	N/A[3]

Abbreviations: Bup = buprenorphine, KY = Kentucky, OH = Ohio, Tele = telemedicine

*Censored because count falls between 1 and 10.

[1]Individuals with no buprenorphine in a given quarter were not included in the denominator for calculating the percent of those in treatment receiving telemedicine or non-telemedicine buprenorphine.

[2]Treatment in next quarter only evaluated for those with treatment in a given quarter.

[3]Patients were not followed past Q4 2020.

© 2023 Hammerslag LR et al. *JAMA Network Open.*

eTable 5. Frequency of Telemedicine Initiations, Relative to All Buprenorphine Initiations, in Kentucky

**Kentucky**	**Q1 2020**		**Q2 2020**		**Q3 2020**		**Q4 2020**	
**% With telemedicine-associated Buprenorphine initiation[1]**	**% With telemedicine**	***p* value**	**% With telemedicine**	***p* value**	**% With telemedicine**	***p* value**	**% With telemedicine**	***p* value**
**Race/Ethnicity**		0.98		0.06		0.20		0.04
Hispanic	0.00%		0.00%		11.76%		11.54%	
Non-Hispanic Black	2.61%		15.18%		8.33%		3.70%	
Non-Hispanic White	2.43%		16.74%		15.35%		11.86%	
Others[2]	2.33%		12.01%		15.75%		11.97%	
**Age group**		0.40		0.57		0.76		0.45
18-34	2.11%		15.71%		14.84%		10.88%	
35-54	2.70%		16.64%		15.37%		12.07%	
55-64	2.19%		18.09%		16.45%		12.87%	
**Sex**		0.56		0.02		0.13		0.62
Male	2.30%		15.06%		14.43%		11.83%	
Female	2.55%		17.67%		16.01%		11.32%	
**Living Area**		0.45		0.11		0.21		0.06
Urban	2.63%		17.41%		14.39%		10.43%	
Rural	2.29%		15.56%		15.71%		12.35%	
Missing Urban/Rural Category	N/A		N/A		N/A		N/A	
**Comorbidities during 60-day lookback**		0.005		0.06		0.17		0.004
Any Mental Health Codes	3.27%		17.72%		16.12%		13.51%	
No Mental Health Codes	1.98%		15.52%		14.65%		10.51%	
**Opioid-related overdose during the 60-day lookback**		0.14		0.16		0.05		0.28
Yes	4.94%		11.32%		9.02%		7.69%	
No	2.38%		16.41%		15.35%		11.67%	
**Total: N[a] (% with telemedicine)**	5,042 (2.42%)		4,341 (16.29%)		4,831 (15.19%)		4,036 (11.60%)	

[1]The N is only reported for the overall total, as a small number of individuals had telemedicine initiation in Q1 2020 and censoring rules prohibit the reporting of stratified counts that are between 1 and 10.

[2]The “Others” race category includes those with a race other than White or Black as well as those with unknown race and ethnicity.

© 2023 Hammerslag LR et al. *JAMA Network Open.*

eTable 6. Frequency of Telemedicine Initiations, Relative to All Buprenorphine Initiations, in Ohio

**Ohio**	**Q1 2020**		**Q2 2020**		**Q3 2020**		**Q4 2020**	
**% With telemedicine-associated Buprenorphine initiation[1]**	**% With telemedicine**	***p* value**	**% With telemedicine**	***p* value**	**% With telemedicine**	***p* value**	**% With telemedicine**	***p* value**
**Race/Ethnicity**		0.33		0.73		0.15		0.006
Hispanic	1.80%		15.07%		9.80%		7.88%	
Non-Hispanic Black	0.59%		14.32%		13.07%		10.65%	
Non-Hispanic White	1.26%		15.38%		15.22%		15.08%	
Others[2]	1.96%		13.25%		16.67%		14.77%	
**Age group**		0.13		0.92		0.29		0.28
18-34	1.50%		15.03%		14.30%		14.26%	
35-54	0.96%		15.41%		15.77%		15.00%	
55-64	1.54%		15.11%		15.16%		11.46%	
**Sex**		0.52		0.08		0.05		0.03
Male	1.17%		14.44%		14.15%		13.53%	
Female	1.35%		16.07%		15.94%		15.56%	
**Living Area**		0.05		0.28		< 0.001		0.06
Urban	1.11%		15.32%		16.06%		15.06%	
Rural	1.80%		15.17%		11.85%		12.50%	
Missing Urban/Rural Category	0.00%		8.45%		13.64%		17.46%	
**Comorbidities during 60-day lookback**		0.58		< 0.001		0.75		< 0.001
Any Mental Health Codes	1.34%		19.06%		15.24%		17.55%	
No Mental Health Codes	1.19%		12.04%		14.81%		11.94%	
**Opioid-related overdose during the 60-day lookback**		0.98		0.71		0.02		0.02
Yes	1.23%		15.95%		10.49%		9.32%	
No	1.26%		15.16%		15.26%		14.71%	
**Total: N[a] (% with telemedicine)**	6,935 (1.25%)		6,131 (15.20%)		6,197 (15.01%)		5,478 (14.48%)	

[1]The N is only reported for the overall total, as a small number of individuals had telemedicine initiation in Q1 2020 and censoring rules prohibit the reporting of stratified counts that are between 1 and 10.

[2]The “Others” race category includes those with a race other than White or Black as well as those with unknown race and ethnicity.

© 2023 Hammerslag LR et al. *JAMA Network Open.*

eTable 7. Percentage of Individuals Achieving 90-Day Retention in Kentucky and Ohio

	Kentucky	Ohio
	**N (% Retained)**	**N (% Retained)**
**Initiation Modality**		
Non-Telemedicine Initiation	7,731 (44.4%)	10,466 (27.9%)
Telemedicine Initiation	1,441 (48.0%)	1,862 (31.6%)
**Quarter**		
Q2	4,341 (46.6%)	6,131 (29.3%)
Q3	4,831 (43.6%)	6,197 (27.7%)
**Race/Ethnicity**		
Hispanic	29 (41.4%)	299 (21.7%)
Non-Hispanic Black	232 (25.4%)	841 (19.5%)
Non-Hispanic White	8,216 (45.8%)	10,586 (29.2%)
Others[1]	695 (42.3%)	602 (32.1%)
**Age group**		
18-34	4,031 (41.9%)	6,237 (24.6%)
35-54	4,722 (46.5%)	5,503 (31.9%)
55-64	419 (57.5%)	588 (37.9%)
**Sex**		
Male	4,812 (43.3%)	6,499 (26.7%)
Female	4,360 (46.8%)	5,829 (30.5%)
**Living Area**		
Urban	3,596 (38.0%)	9,159 (27.2%)
Rural	5,576 (49.5%)	3,010 (32.6%)
Missing Urban/Rural Category	N/A	159 (23.3%)
**Comorbidities during 60-day lookback**		
Any Mental Health Codes	3,298 (44.9%)	5,561 (27.4%)
No Mental Health Codes	5,874 (45.0%)	6,767 (29.4%)
**Opioid-related overdose during the 60-day lookback**		
Yes	228 (29.8%)	625 (16.8%)
No	8,944 (45.4%)	11,703 (29.1%)
**Total, N**	9,172 (45.0%)	12,328 (28.5%)

[1]The “Others” race category includes those with a race other than White or Black as well as those with unknown race and ethnicity.

© 2023 Hammerslag LR et al. *JAMA Network Open.*

eTable 8. Sensitivity Analysis Examining Percentage of Individuals Achieving 30-Day Retention in Kentucky and Ohio

	Kentucky	Ohio
	**N (% Retained)**	**N (% Retained)**
**Initiation Modality**		
Non-Telemedicine Initiation	7,731 (68.7%)	10,466 (47.1%)
Telemedicine Initiation	1,441 (74.6%)	1,862 (53.3%)
**Quarter**		
Q2	4,341 (70.5%)	6,131 (49.8%)
Q3	4,831 (68.8%)	6,197 (46.3%)
**Race/Ethnicity**		
Hispanic	29 (69.0%)	299 (45.5%)
Non-Hispanic Black	232 (54.7%)	841 (37.8%)
Non-Hispanic White	8,216 (70.1%)	10,586 (48.8%)
Others[1]	695 (69.1%)	602 (50.8%)
**Age group**		
18-34	4,031 (66.7%)	6,237 (44.3%)
35-54	4,722 (71.5%)	5,503 (51.2%)
55-64	419 (76.8%)	588 (58.7%)
**Sex**		
Male	4,812 (68.5%)	6,499 (46.6%)
Female	4,360 (70.9%)	5,829 (49.7%)
**Living Area**		
Urban	3,596 (62.6%)	9,159 (46.7%)
Rural	5,576 (74.2%)	3,010 (52.2%)
Missing Urban/Rural Category	N/A	159 (48.4%)
**Comorbidities during 60-day lookback**		
Any Mental Health Codes	3,298 (68.5%)	5,561 (47.7%)
No Mental Health Codes	5,874 (70.3%)	6,767 (48.4%)
**Opioid-related overdose during the 60-day lookback**		
Yes	228 (57.9%)	625 (37.9%)
No	8,944 (69.9%)	11,703 (48.6%)
**Total, N**	9,172 (69.6%)	12,328 (48.1%)

[1]The “Others” race category includes those with a race other than White or Black as well as those with unknown race and ethnicity.

© 2023 Hammerslag LR et al. *JAMA Network Open.*

eTable 9. Percentage of Individuals With an Opioid-Related Overdose During the 90-Day Follow-up Period in Kentucky and Ohio

	Kentucky	Ohio
**% of Total**	**N (% with overdose)**	**N (% with overdose)**
**Initiation Modality**		
Non-Telemedicine Initiation	7,731 (1.8%)	10,466 (3.6%)
Telemedicine Initiation	1,441 (1.5%)	1,862 (3.8%)
**Quarter**		
Q2	4,341 (2.0%)	6,131 (3.9%)
Q3	4,831 (1.6%)	6,197 (3.4%)
**Race/Ethnicity**		
Hispanic	29 (0.0%)	299 (5.0%)
Non-Hispanic Black	232 (5.2%)	841 (4.2%)
Non-Hispanic White	8,216 (1.7%)	10,586 (3.5%)
Others[1]	695 (1.9%)	602 (3.5%)
**Age group**		
18-34	4,031 (2.5%)	6,237 (4.0%)
35-54	4,722 (1.2%)	5,503 (3.2%)
55-64	419 (*)	588 (3.1%)
**Sex**		
Male	4,812 (2.1%)	6,499 (4.4%)
Female	4,360 (1.4%)	5,829 (2.7%)
**Living Area**		
Urban	3,596 (3.1%)	9,159 (3.9%)
Rural	5,576 (1.0%)	3,010 (2.7%)
Missing Urban/Rural Category	N/A	151 (*)
**Comorbidities during 60-day lookback**		
Any Mental Health Codes	3,298 (2.0%)	5,561 (4.6%)
No Mental Health Codes	5,874 (1.7%)	6,767 (2.8%)
**Opioid-related overdose during the 60-day lookback**		
Yes	228 (10.1%)	625 (13.4%)
No	8,944 (1.6%)	11,703 (3.1%)
**Total, N**	9,172 (1.8%)	12,328 (3.6%)

*Censored because count with outcome falls between 1 and 10.

[1]The "Others" race category includes those with a race other than White or Black as well as those with unknown race and ethnicity.

© 2023 Hammerslag LR et al. *JAMA Network Open.*

eTable 10. Sensitivity Analysis Examining Percentage of Individuals With an Opioid-Related Overdose During the 30-Day Follow-up Period in Kentucky and Ohio

% of Total	Kentucky<br>N (% with overdose)	Ohio<br>N (% with overdose)
**Initiation Modality**		
Non-Telemedicine Initiation	7,731 (0.8%)	10,466 (1.5%)
Telemedicine Initiation	1,441 (*)	1,862 (1.2%)
**Quarter**		
Q2	4,341 (0.9%)	6,131 (1.5%)
Q3	4,831 (0.6%)	6,197 (1.4%)
**Race/Ethnicity**		
Hispanic	29 (0.0%)	299 (*)
Non-Hispanic Black	232 (*)	841 (1.7%)
Non-Hispanic White	8,216 (0.8%)	10,586 (1.4%)
Others[1]	695 (*)	593 (*)
**Age group**		
18-34	4,031 (0.9%)	6,237 (1.6%)
35-54	4,722 (0.6%)	5,503 (1.2%)
55-64	419 (*)	582 (*)
**Sex**		
Male	4,812 (0.8%)	6,499 (1.8%)
Female	4,360 (0.6%)	5,829 (1.0%)
**Living Area**		
Urban	3,596 (1.3%)	9,159 (1.5%)
Rural	5,576 (0.4%)	3,010 (1.2%)
Missing Urban/Rural Category	N/A	157 (*)
**Comorbidities during 60-day lookback**		
Any Mental Health Codes	3,298 (0.9%)	5,561 (1.8%)
No Mental Health Codes	5,874 (0.6%)	6,767 (1.1%)
**Opioid-related overdose during the 60-day lookback**		
Yes	228 (5.3%)	625 (6.9%)
No	8,944 (0.6%)	11,703 (1.1%)
**Total, N**	9,172 (0.7%)	12,328 (1.4%)

*Censored because count with outcome falls between 1 and 10.

[1]The "Others" race category includes those with a race other than White or Black as well as those with unknown race and ethnicity.

© 2023 Hammerslag LR et al. *JAMA Network Open.*

eFigure 1. Adjusted Odds of Retention Over the First 30 Days After Initiation in Kentucky (A) or Ohio (B)

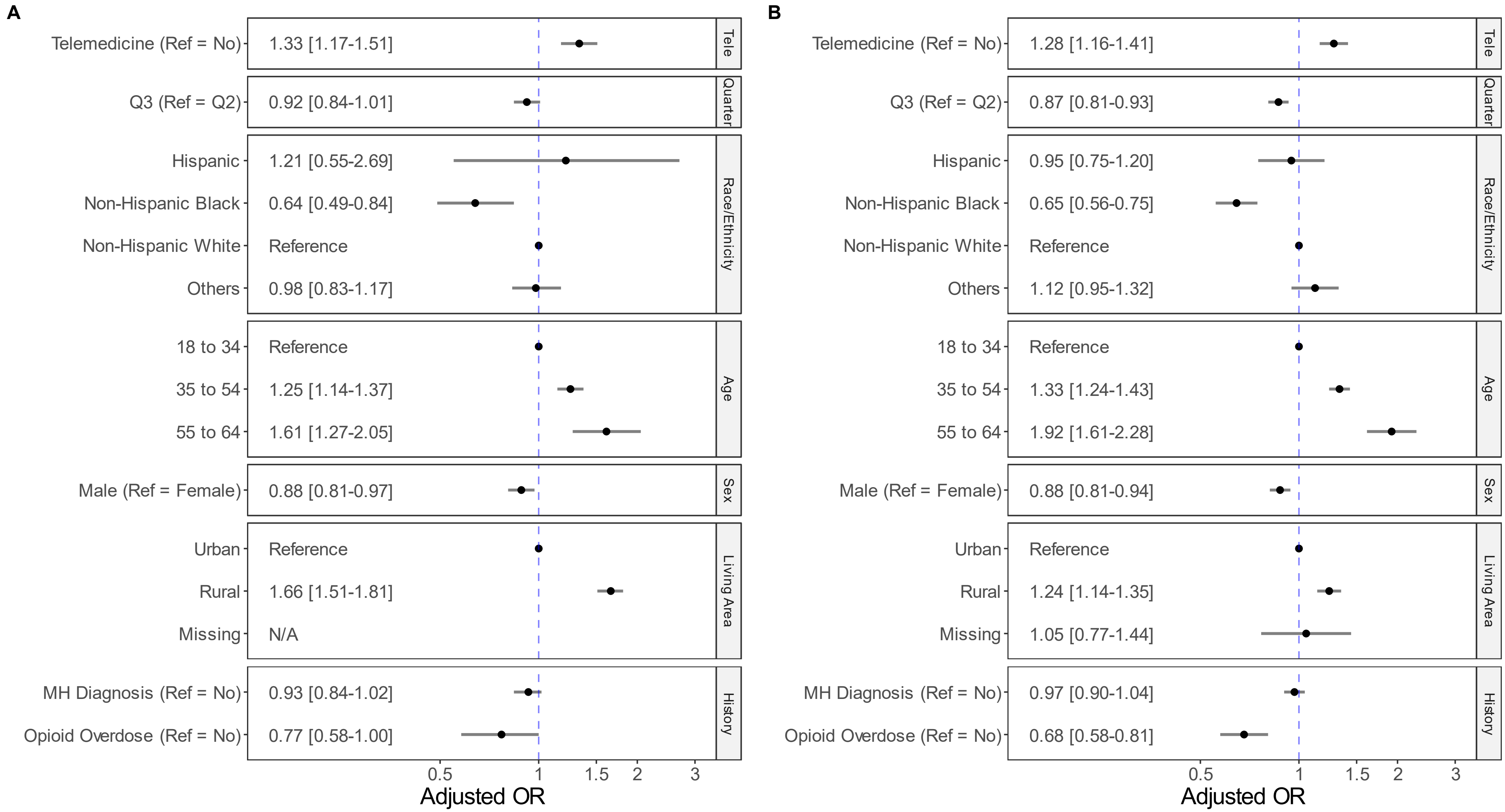

Analysis limited to those with buprenorphine initiations in Q2 or Q3 2020.

Abbreviations: Telemedicine = telemedicine initiation, Q = quarter, MH = mental health, OR = odds ratio.

Note: The “Others” race category includes those with a race other than White or Black as well as those with unknown race and ethnicity.

© 2023 Hammerslag LR et al. *JAMA Network Open.*

eFigure 2. Adjusted Odds of Opioid-Related Nonfatal Overdose in the First 30 Days After Initiation in Kentucky (A) or Ohio (B)

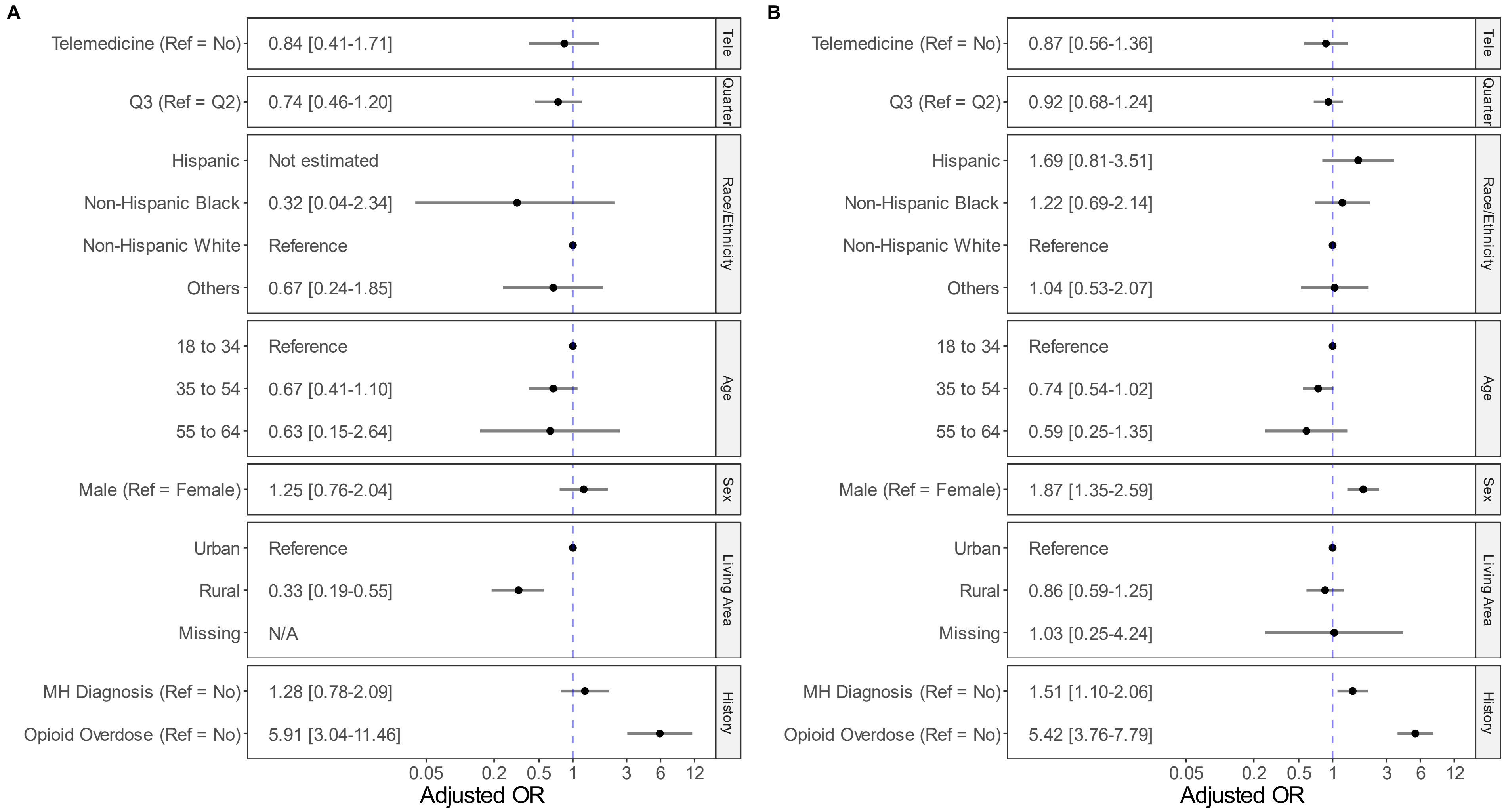

Analysis limited to those with buprenorphine initiations in Q2 or Q3 2020.

Abbreviations: Telemedicine = telemedicine initiation, Q = quarter, MH = mental health, OR = odds ratio.

Note: The "Others" race category includes those with a race other than White or Black as well as those with unknown race and ethnicity.

© 2023 Hammerslag LR et al. *JAMA Network Open.*

eAppendix 1. National Drug Codes (NDC) Used to Identify Transmucosal Buprenorphine FDA-Approved for the Treatment of OUD

62756045983, 71335095002, 00093537856, 71335095001, 55700030230, 12496127802, 60687048121, 00378092393, 60687048111, 00054017613, 71335095003, 35356055530, 35356055530, 00228315603, 49999063830, 50383092493, 68308020230, 42858050103, 60687049221, 71335115401, 55700030330, 63874117303, 71335115403, 71335115402, 68308020830, 43063075306, 71335116301, 35356055630, 63629409201, 49999063930, 42858050203, 12496131002, 00378092493, 54569657800, 60687049211, 50383093093, 53217024630, 50090292400, 71335116303, 00054017713, 71335116302, 62756046083, 00093537956, 68258299103, 00228315303, 54123090730, 54123091430, 00490005100, 49999039515, 16729054910, 60429058630, 63629402801, 00054018813, 62756096983, 49999039530, 00228315473, 60429058633, 65162041603, 68071151003, 00490005160, 71335129601, 54868575000, 00904700906, 63874108503, 16590066605, 49999039507, 00406192303, 00093572056, 00228315403, 55700090130, 00490005190, 55700018430, 16590066630, 50383029493, 68258299903, 12496128302, 42291017430, 55700018430, 62175045232, 50268014411, 54569549600, 50268014415, 00490005130, 52959074930, 00406800503, 54123092930, 54123095730, 63629403401, 54868570703, 43063018430, 53217013830, 50383028793, 16590066705, 62756097083, 52959030430, 00406192403, 60429058730, 54868570702, 00228315567, 68071138003, 23490927003, 62175045832, 00904701006, 00228315573, 23490927006, 55045378403, 16590066730, 23490927009, 66336001630, 00228315503, 00054018913, 35356000430, 54868570704, 35356000407, 16729055010, 43063018407, 54569573902, 42291017530, 54569640800, 00406802003, 54868570700, 60429058733, 16590066790, 00093572156, 12496130602, 54569573901, 63629403402, 50268014511, 50268014515, 54569573900, 63629403403, 54868570701, 65162041503, 63874108403, 54123098630, 54123011430, 52427069203, 12496120203, 52427069211, 43598057930, 47781035511, 00378876516, 00378876593, 43598057901, 00781721664, 12496120201, 47781035503, 00781721606, 00378876693, 00781722706, 12496120403, 12496120401, 52427069403, 52427069411, 47781035603, 00378876616, 00781722764, 43598058001, 47781035611, 43598058030, 12496120801, 12496120803, 00781723864, 52427069811, 54569639900, 43598058201, 47781035703, 47781035711, 43598058230, 00378876716, 00781723806, 55700014730, 52427069803, 00378876793, 00781724964, 47781035803, 12496121201, 47781035811, 52427071203, 43598058130, 12496121203, 00781724906, 52427071211, 00378876816, 43598058101, 00378876893, 59385001230, 59385001201, 59385001401, 59385001430, 59385001601, 59385001630

© 2023 Hammerslag LR et al. *JAMA Network Open.*

eAppendix 2. Diagnosis Codes for Mental Health Conditions (the Medicaid Outcomes Distributed Research Network (MODRN) et al, 2021)

Include ICD-10-CM diagnosis codes with any of the following in the first three positions:

Schizophrenia, schizotypal, delusional and other non-mood psychotic disorders: F20-F29
Mood disorders: F30-F39
Anxiety disorders and OCD: F40-F42
PTSD: F431
Eating disorders: F50
Specific personality disorders: F60
Attention deficit and conduct disorders: F90-91

© 2023 Hammerslag LR et al. *JAMA Network Open*.

eAppendix 3. Diagnosis Codes for Opioid-Related Overdoses (Poisonings)

Include ICD-10-CM diagnosis codes meeting the following guidelines:

1. Any of the following in the first 5 positions:
   T40.0X–T40.4X, T40.60, T40.69

AND

2. a 6th character of
   1: Accidental (unintentional)
   2: Intentional self-harm
   3: Assault
   4: Undetermined intent

AND

3. a 7th character of
   A
   OR
   missing

© 2023 Hammerslag LR et al. *JAMA Network Open*.

eAppendix 4. Protocol for Identifying Telemedicine Visits

For a given Bup Rx, a fill is tele-associated if all of the following guidelines are met:

1. Service date between (Bup Rx dispensing date – 7) and (Bup Rx dispensing date)

AND

2. OUD diagnosis: First or second diagnosis code field meets the following guidelines:
   a. First three characters are F11
   AND
   b. Fourth character is either 1 or 2
   AND
   c. Fifth character is not 1

AND

3. Any one of the following Modifier, Place of Service, Procedure or Revenue codes (American Academy of Family Physicians, 2020; Barsky et al., 2022; Centers for Medicare & Medicaid Services, 2020).

Code	Code type	Source of definition
2	Place of Service	AAFP, KY-specific
95	Modifier	AAFP, CMS, Barsky et al., 2022
780	Revenue	KY-specific
98966	Procedure	CMS, Barsky et al., 2022
98967	Procedure	CMS, Barsky et al., 2022
98968	Procedure	CMS, Barsky et al., 2022
99441	Procedure	AAFP, CMS, Barsky et al., 2022
99442	Procedure	AAFP, CMS, Barsky et al., 2022
99443	Procedure	AAFP, CMS, Barsky et al., 2022
G2025	Procedure	Barsky 2022
GQ	Modifier	OH-specific, CMS, Barsky et al., 2022
GT	Modifier	AAFP, CMS, Barsky et al., 2022

© 2023 Hammerslag LR et al. *JAMA Network Open.*
