# Supplementary material for: Telemedicine Buprenorphine Initiation and Retention in Opioid Use Disorder Treatment for Medicaid Enrollees
Source: JAMA Netw Open. 2023 Oct 18;6(10):e2336914. doi: 10.1001/jamanetworkopen.2023.36914 (PMC10585416; doi:10.1001/jamanetworkopen.2023.36914)
Supplement: Supplement 2. — Data Sharing Statement [file jamanetwopen-e2336914-s002.pdf]

Data Sharing Statement

Hammerslag. Telemedicine Buprenorphine Initiation and Retention in Opioid Use Disorder Treatment for Medicaid Enrollees. *JAMA Netw Open*. Published October 13, 2023. doi:10.1001/jamanetworkopen.2023.36914

Data
Data available: No

Additional Information
Explanation for why data not available: Because this study uses Medicaid data, we are bound by data use agreements. Only aggregate counts with more than 10 individuals can be shared.
